# Supplementary figures and images for: Nicotine enhances the stemness and tumorigenicity in intestinal stem cells via Hippo-YAP/TAZ and Notch signal pathway
Source: eLife. 2025 Jan 3;13:RP95267. doi: 10.7554/eLife.95267 (PMC11698494; doi:10.7554/eLife.95267)

Figure 3D      $\alpha 7$ -nAChR

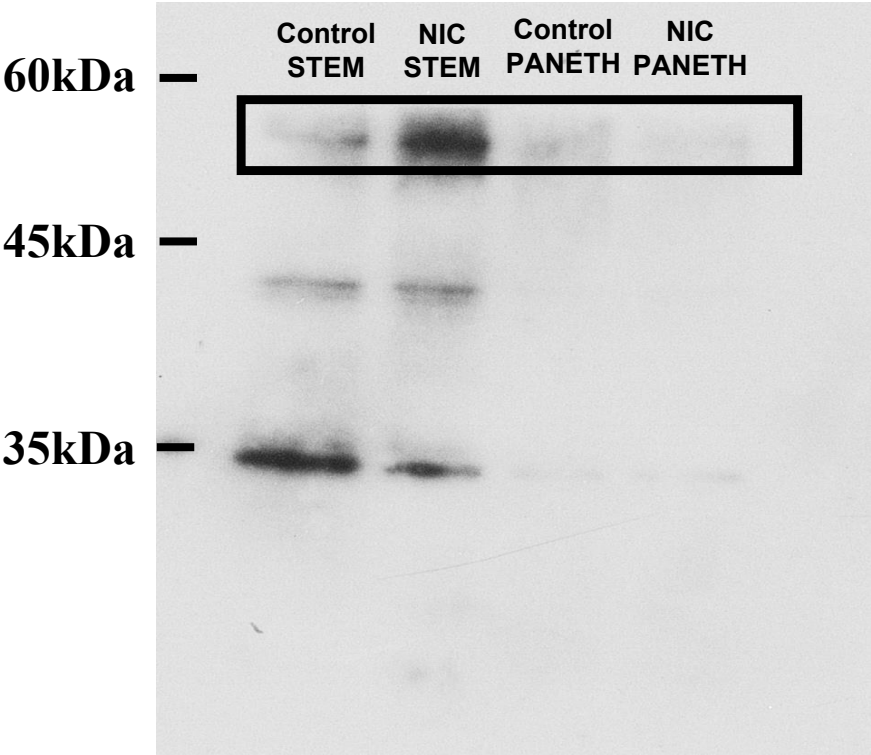

Supplement: Figure 3—source data 2. [file elife-95267-fig3-data2.zip › Figure 3 Source Data 2/Figure 3D ╬▒7 with Labelling.pdf]

**Figure 3D      $\beta$ -Actin**

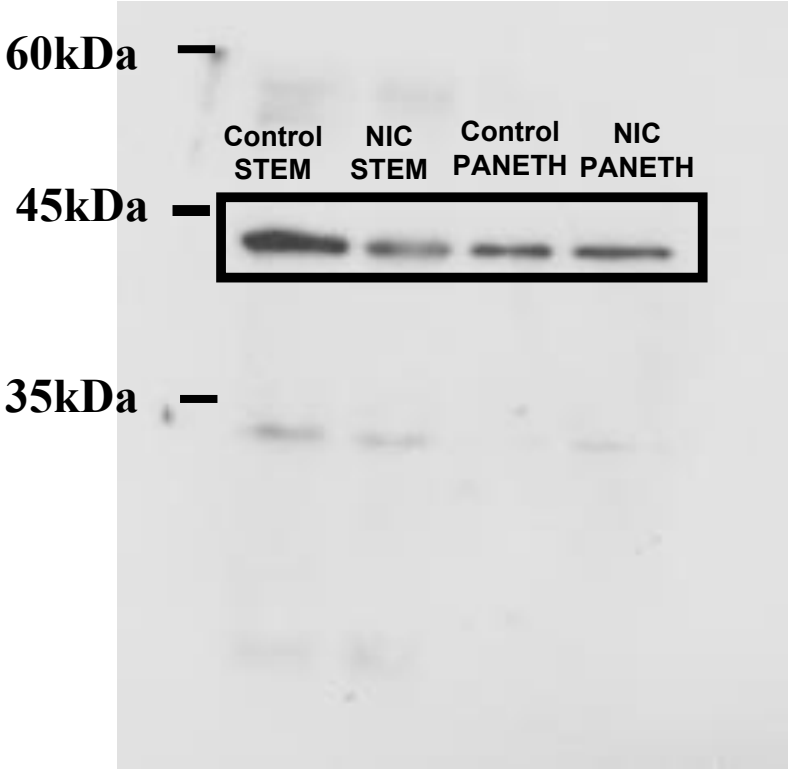

Supplement: Figure 3—source data 2. [file elife-95267-fig3-data2.zip › Figure 3 Source Data 2/Figure 3D ╬▓-Actin with Labelling.pdf]

**Figure 3D**     $\alpha 7$ -nAChR

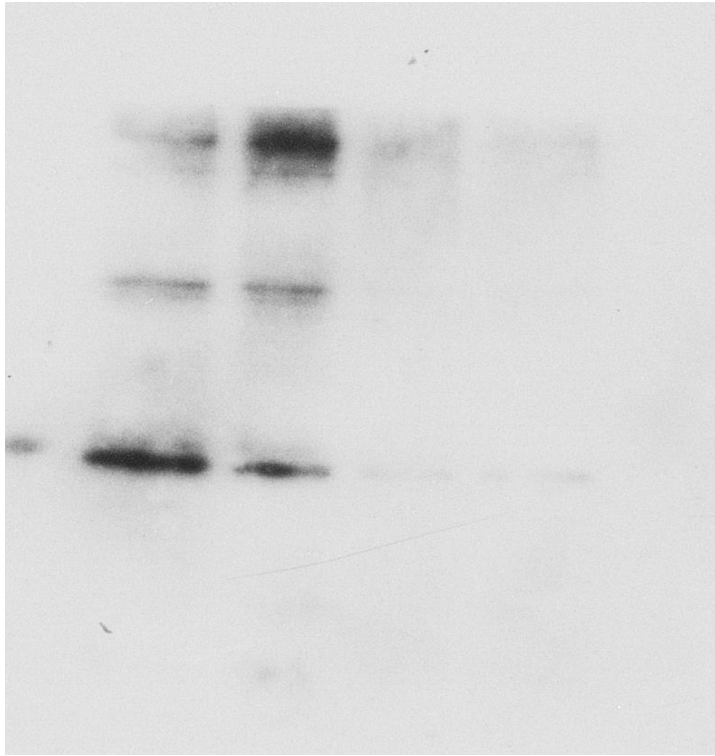

Supplement: Figure 3—source data 3. [file elife-95267-fig3-data3.zip › Figure 3 Source Data 3/Figure 3D ╬▒7 Raw Data.pdf]

**Figure 3D**      **$\beta$ -Actin**

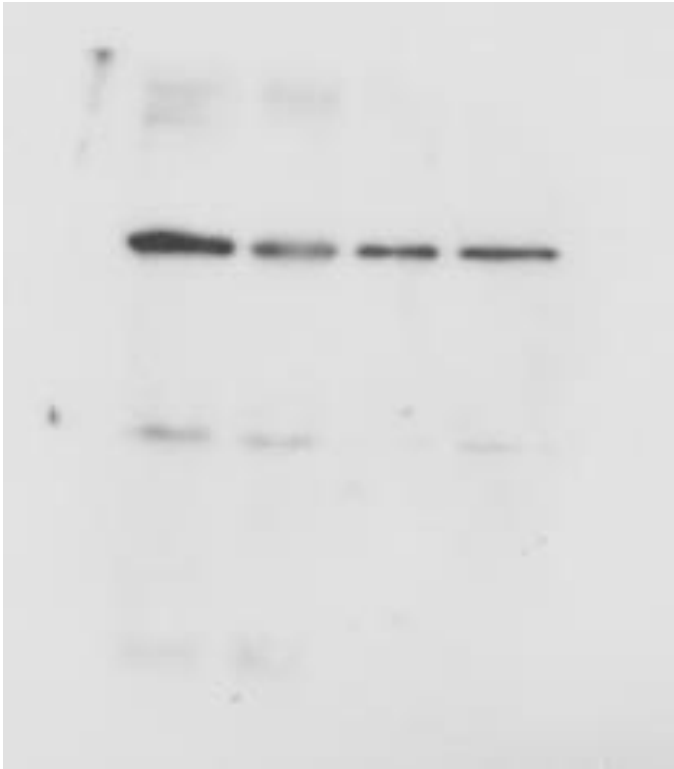

Supplement: Figure 3—source data 3. [file elife-95267-fig3-data3.zip › Figure 3 Source Data 3/Figure 3D ╬▓-Actin Raw Data.pdf]

Figure 4C    TAZ

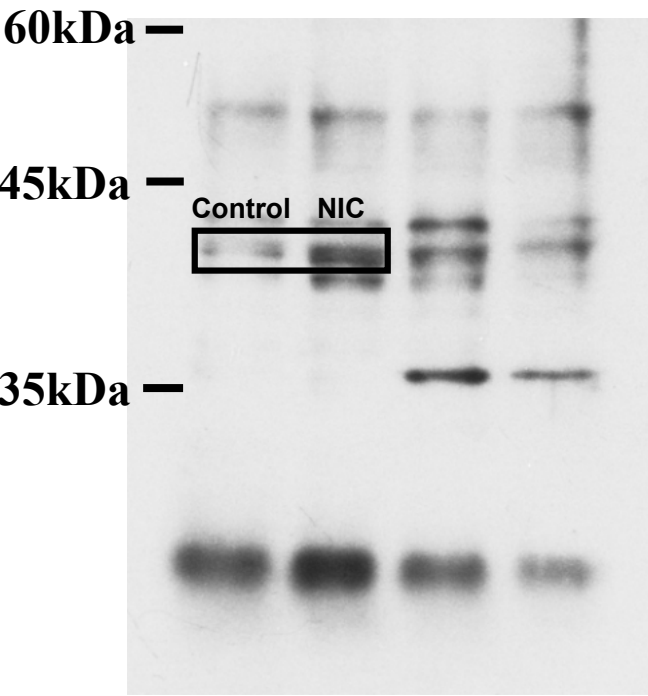

Supplement: Figure 4—source data 2. [file elife-95267-fig4-data2.zip › Figure 4-Source Data 2/Figure 4C with Labelling/Figure 4C TAZ with Labelling.pdf]

**Figure 4C YAP**

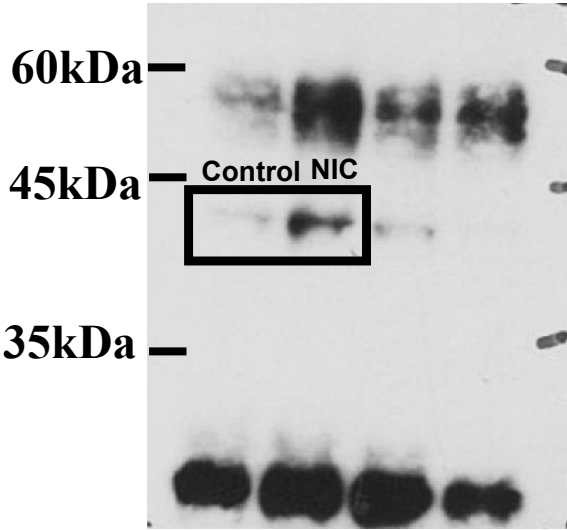

Supplement: Figure 4—source data 2. [file elife-95267-fig4-data2.zip › Figure 4-Source Data 2/Figure 4C with Labelling/Figure 4C YAP with Labelling.pdf]

Figure 4C  $\beta$ -Actin

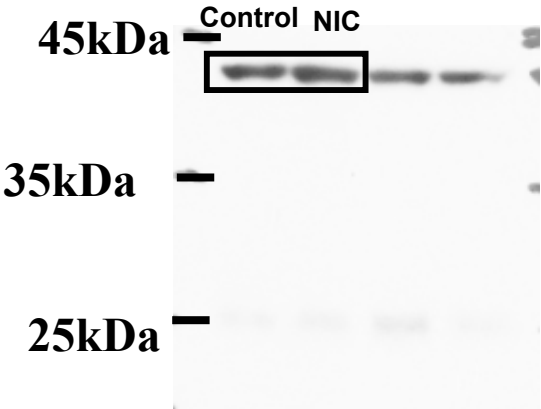

Supplement: Figure 4—source data 2. [file elife-95267-fig4-data2.zip › Figure 4-Source Data 2/Figure 4C with Labelling/Figure 4C ╬▓-Actin with Labelling.pdf]

**Figure 4E**    **Hes5**

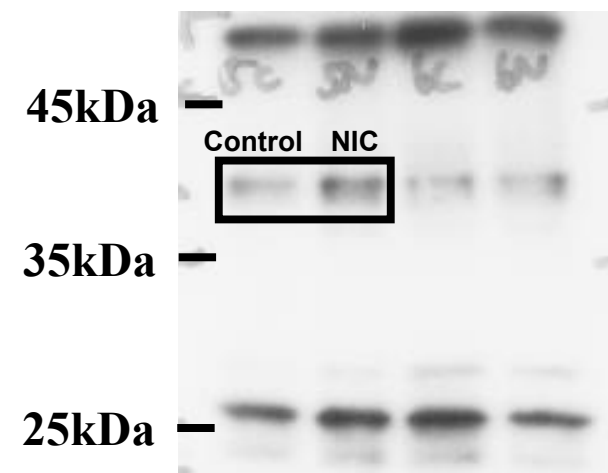

Supplement: Figure 4—source data 2. [file elife-95267-fig4-data2.zip › Figure 4-Source Data 2/Figure 4E with Labelling/Figure 4E Hes5 with Labelling.pdf]

**Figure 4E Jagged1**

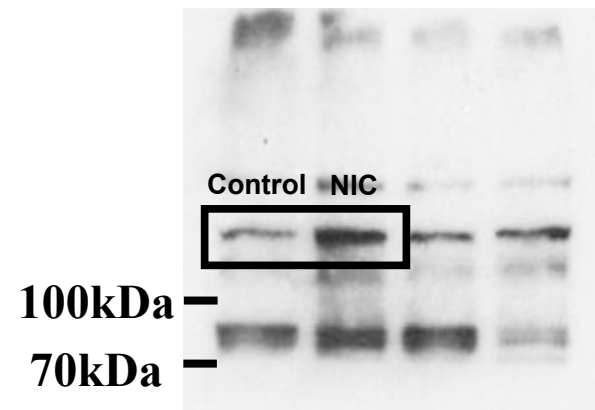

Supplement: Figure 4—source data 2. [file elife-95267-fig4-data2.zip › Figure 4-Source Data 2/Figure 4E with Labelling/Figure 4E Jagged1 with Labelling.pdf]

**Figure 4E Jagged2**

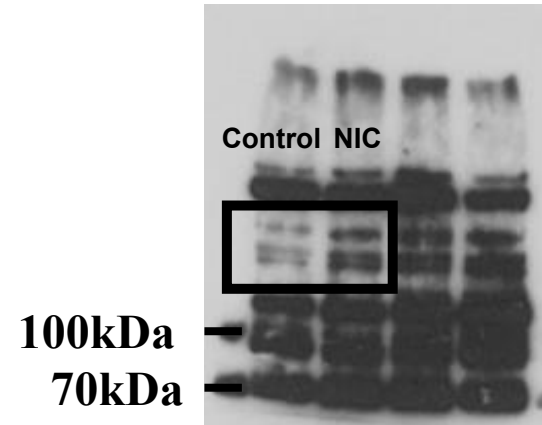

Supplement: Figure 4—source data 2. [file elife-95267-fig4-data2.zip › Figure 4-Source Data 2/Figure 4E with Labelling/Figure 4E Jagged2 with Labelling.pdf]

**Figure 4E Notch1**

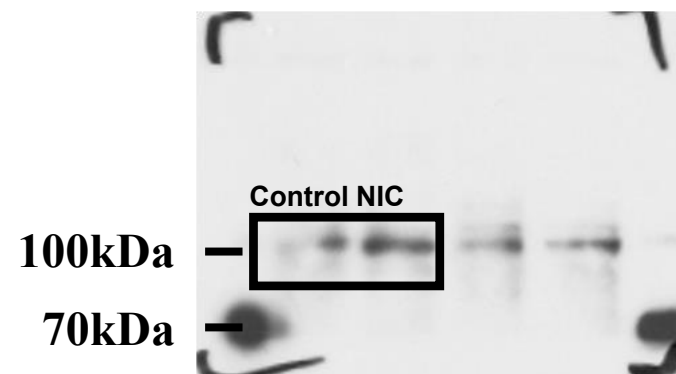

Supplement: Figure 4—source data 2. [file elife-95267-fig4-data2.zip › Figure 4-Source Data 2/Figure 4E with Labelling/Figure 4E Notch1 with Labelling.pdf]

**Figure 4E     β-Actin**

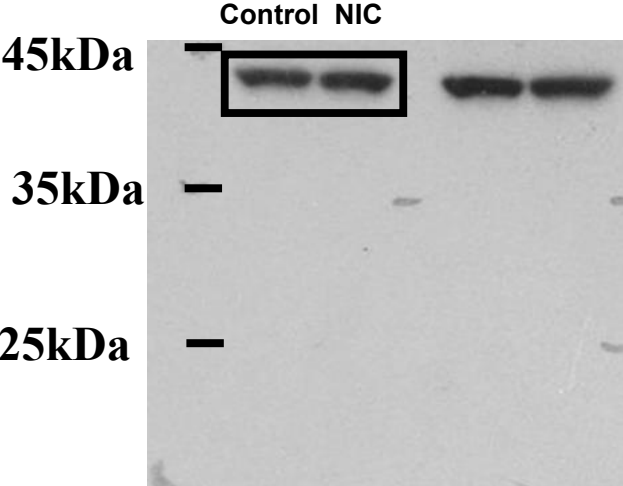

Supplement: Figure 4—source data 2. [file elife-95267-fig4-data2.zip › Figure 4-Source Data 2/Figure 4E with Labelling/Figure 4E ╬▓-Actin with Labelling.pdf]

**Figure 4C YAP**

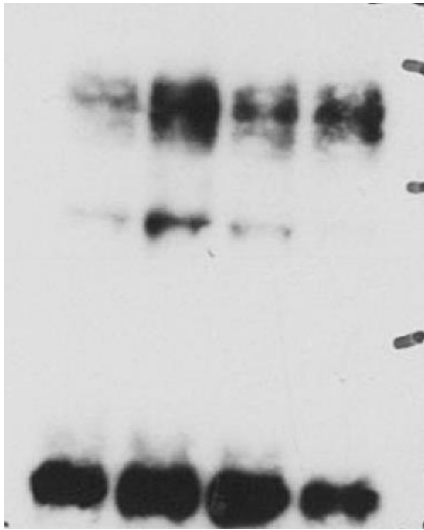

Supplement: Figure 4—source data 3. [file elife-95267-fig4-data3.zip › Figure 4-Source Data 3/Figure 4C Raw Data/Figure 4C YAP Raw Data.pdf]

**Figure 4C  $\beta$ -Actin**

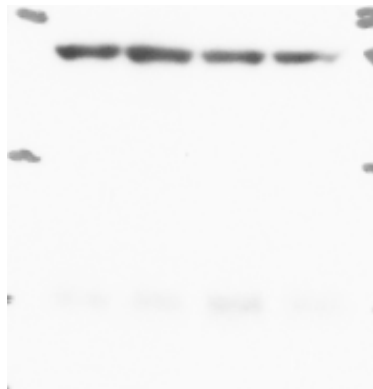

Supplement: Figure 4—source data 3. [file elife-95267-fig4-data3.zip › Figure 4-Source Data 3/Figure 4C Raw Data/Figure 4C ╬▓-Actin Raw Data.pdf]

**Figure 4C    TAZ**

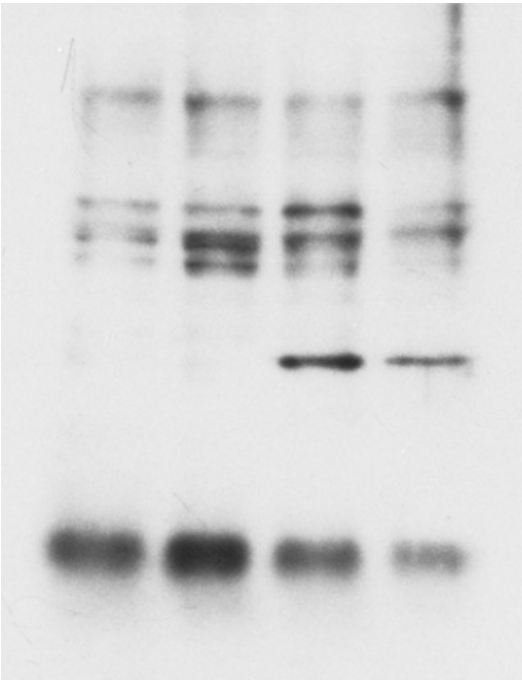

Supplement: Figure 4—source data 3. [file elife-95267-fig4-data3.zip › Figure 4-Source Data 3/Figure 4C Raw Data/Figure 4C TAZ Raw Data.pdf]

**Figure 4E Jagged1**

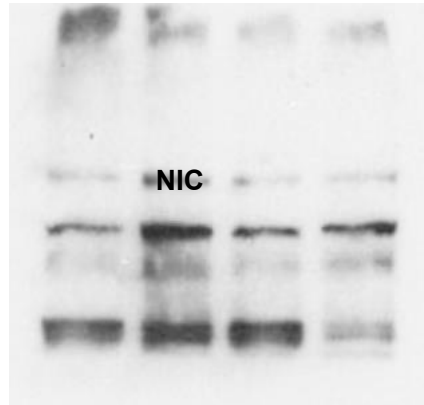

Supplement: Figure 4—source data 3. [file elife-95267-fig4-data3.zip › Figure 4-Source Data 3/Figure 4E Raw Data/Figure 4E Jagged1 Raw Data.pdf]

**Figure 4E** Notch1

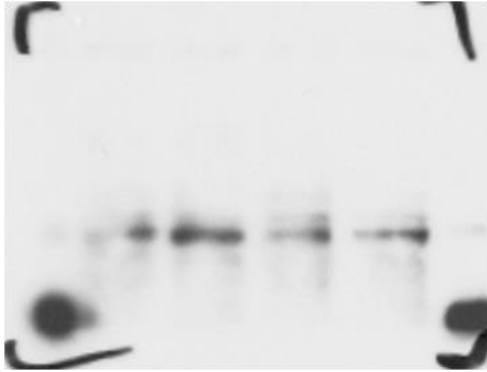

Supplement: Figure 4—source data 3. [file elife-95267-fig4-data3.zip › Figure 4-Source Data 3/Figure 4E Raw Data/Figure 4E Notch1 Raw Data.pdf]

**Figure 4E**      **$\beta$ -Actin**

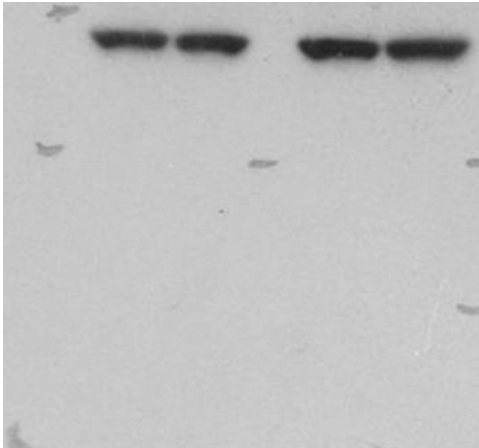

Supplement: Figure 4—source data 3. [file elife-95267-fig4-data3.zip › Figure 4-Source Data 3/Figure 4E Raw Data/Figure 4E ╬▓-Actin Raw Data.pdf]

**Figure 4E**    **Hes5**

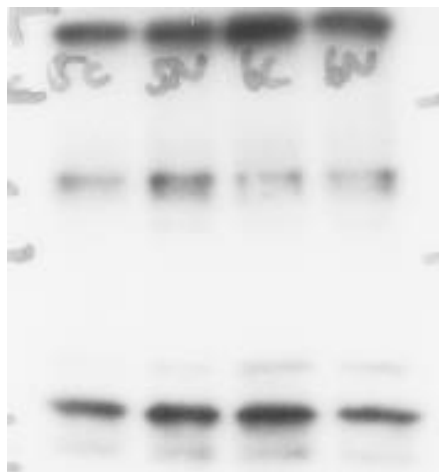

Supplement: Figure 4—source data 3. [file elife-95267-fig4-data3.zip › Figure 4-Source Data 3/Figure 4E Raw Data/Figure 4E Hes5 Raw Data.pdf]

**Figure 4E Jagged2**

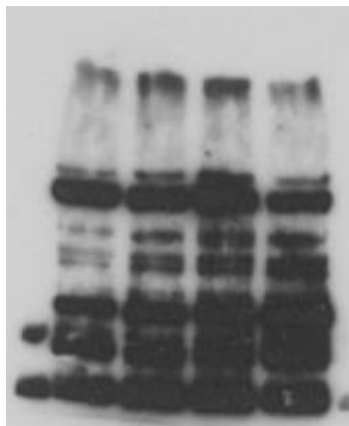

Supplement: Figure 4—source data 3. [file elife-95267-fig4-data3.zip › Figure 4-Source Data 3/Figure 4E Raw Data/Figure 4E Jagged2 Raw Data.pdf]

**Figure 4-Figure Supplemental 1**      **p-S6**

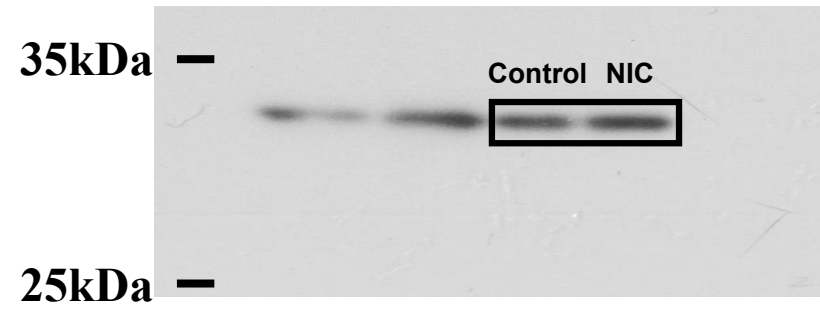

Supplement: Figure 4—figure supplement 1—source data 2. [file elife-95267-fig4-figsupp1-data2.zip › Figure 4-Supplemental Figure 1 Source Data 2/Figure 4-Figure Supplemental 1 p-S6 with Labellling.pdf]

**Figure 4-Figure Supplemental 1     S6**

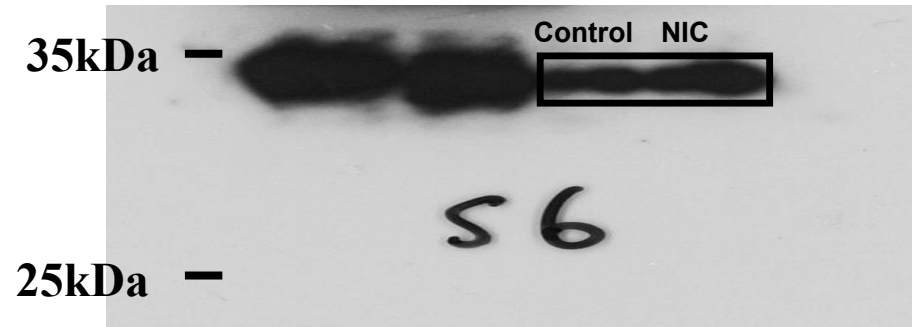

Supplement: Figure 4—figure supplement 1—source data 2. [file elife-95267-fig4-figsupp1-data2.zip › Figure 4-Supplemental Figure 1 Source Data 2/Figure 4-Figure Supplemental 1 S6 with Labelling.pdf]

**Figure 4-Figure Supplemental 1**      **p-p38**

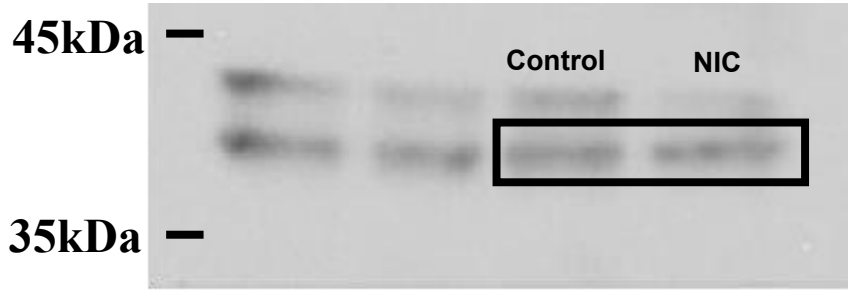

Supplement: Figure 4—figure supplement 1—source data 2. [file elife-95267-fig4-figsupp1-data2.zip › Figure 4-Supplemental Figure 1 Source Data 2/Figure 4-Figure Supplemental 1 p-p38 with Labelling.pdf]

**Figure 4-Figure Supplemental 1**      **p38**

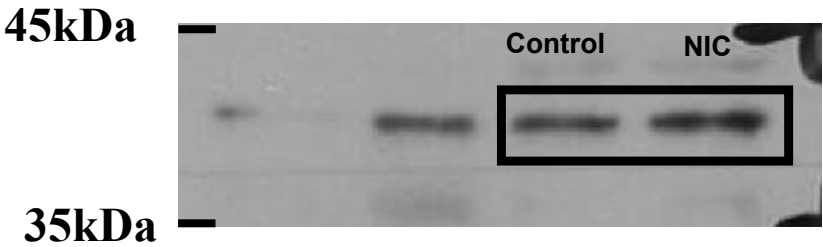

Supplement: Figure 4—figure supplement 1—source data 2. [file elife-95267-fig4-figsupp1-data2.zip › Figure 4-Supplemental Figure 1 Source Data 2/Figure 4-Figure Supplemental 1 p38 with Labelling.pdf]

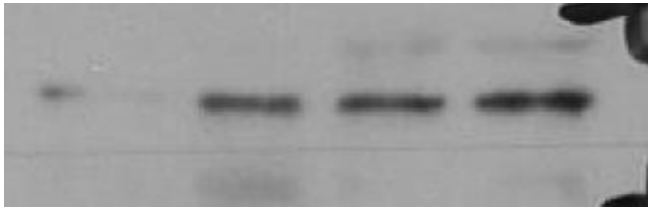

Supplement: Figure 4—figure supplement 1—source data 3. [file elife-95267-fig4-figsupp1-data3.zip › Figure 4-Supplemental Figure 1 Source Data 3/Figure 4-Figure Supplemental 1 p38 Raw Data.pdf]

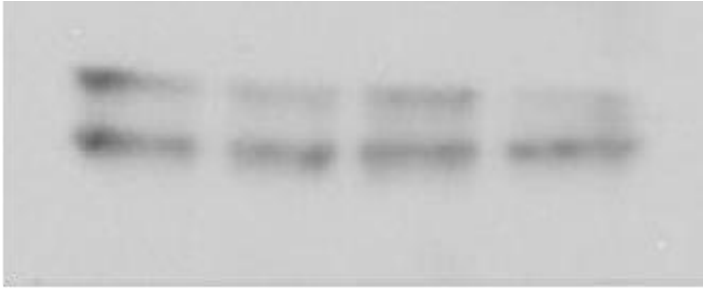

Supplement: Figure 4—figure supplement 1—source data 3. [file elife-95267-fig4-figsupp1-data3.zip › Figure 4-Supplemental Figure 1 Source Data 3/Figure 4-Figure Supplemental 1 p-p38 Raw Data.pdf]

**Figure 4-Figure Supplemental 1 S6**

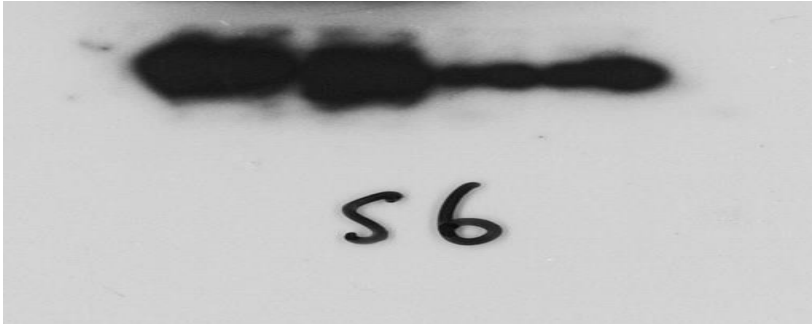

Supplement: Figure 4—figure supplement 1—source data 3. [file elife-95267-fig4-figsupp1-data3.zip › Figure 4-Supplemental Figure 1 Source Data 3/Figure 4-Figure Supplemental 1 S6 Raw Data.pdf]

**Figure 4B Supplemental p-S6**

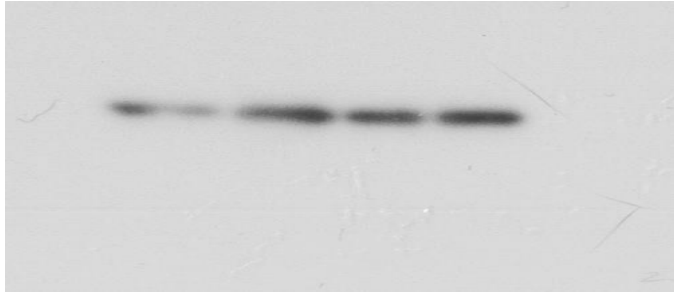

Supplement: Figure 4—figure supplement 1—source data 3. [file elife-95267-fig4-figsupp1-data3.zip › Figure 4-Supplemental Figure 1 Source Data 3/Figure 4-Figure Supplemental 1 p-S6 Raw Data.pdf]

**Figure6B      Hes5**

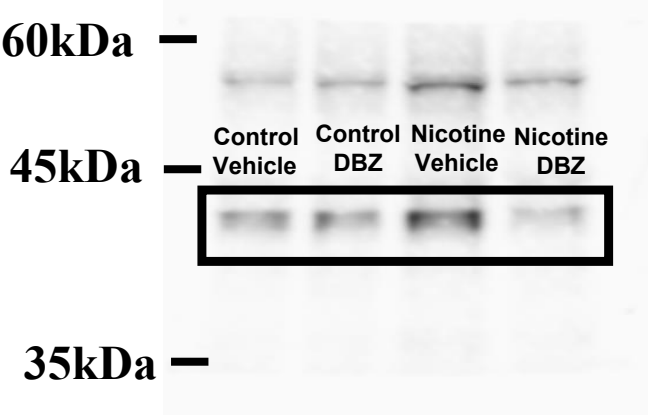

Supplement: Figure 6—source data 2. [file elife-95267-fig6-data2.zip › Figure 6 Source Data 2/Figure 6B Hes5 with Labelling.pdf]

**Figure6B    TAZ**

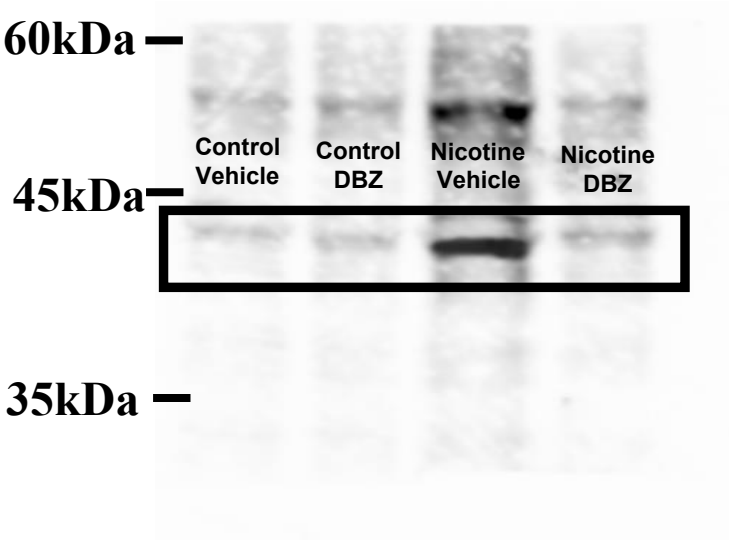

Supplement: Figure 6—source data 2. [file elife-95267-fig6-data2.zip › Figure 6 Source Data 2/Figure 6B TAZ with Labelling.pdf]

**Figure 6B**     **YAP**

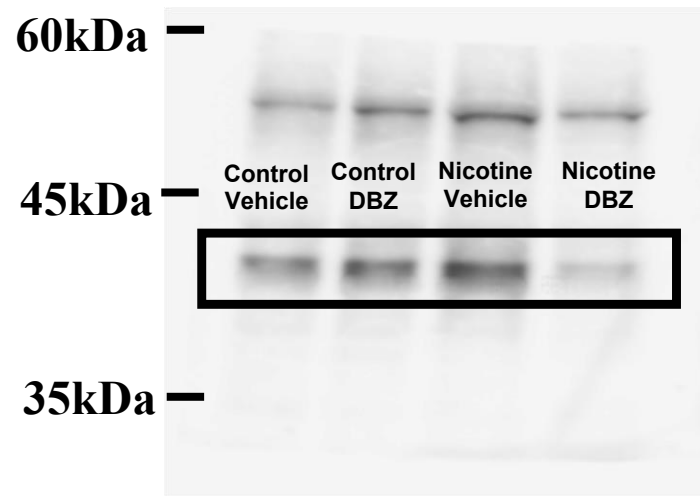

Supplement: Figure 6—source data 2. [file elife-95267-fig6-data2.zip › Figure 6 Source Data 2/Figure 6B YAP with Labelling.pdf]

**Figure 6B**       **$\beta$ -Actin**

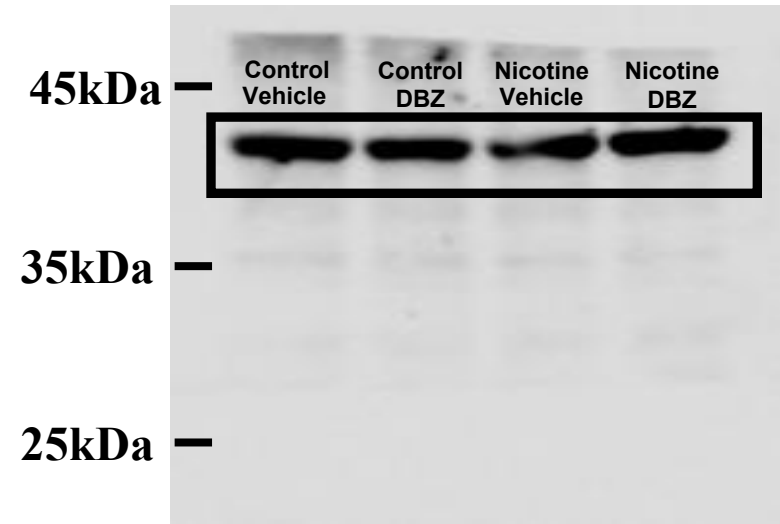

Supplement: Figure 6—source data 2. [file elife-95267-fig6-data2.zip › Figure 6 Source Data 2/Figure 6B ╬▓-Actin with Labelling.pdf]

**Figure6B**      **Hes5**

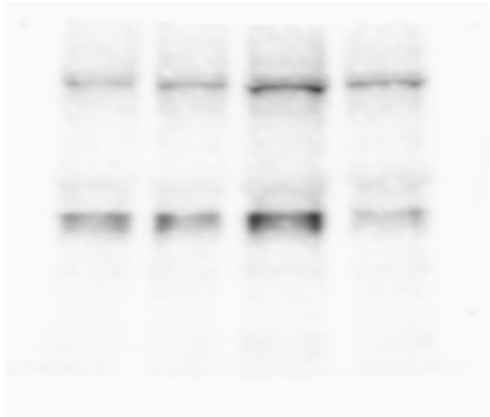

Supplement: Figure 6—source data 3. [file elife-95267-fig6-data3.zip › Figure 6 Source Data 3/Figure 6B Hes5 Raw Data.pdf]

**Figure 6B**       **$\beta$ -Actin**

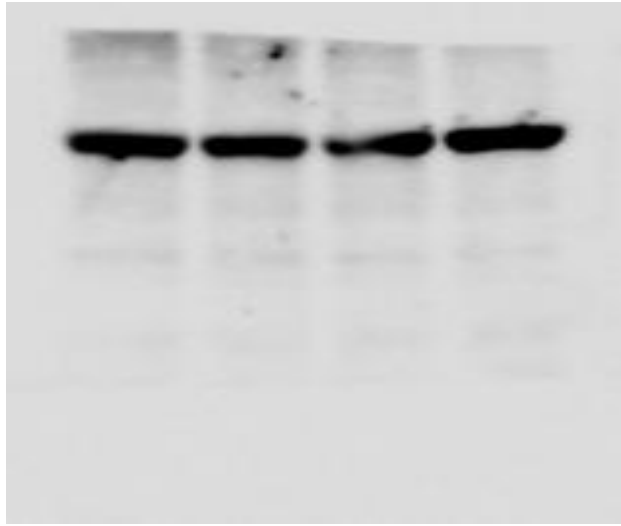

Supplement: Figure 6—source data 3. [file elife-95267-fig6-data3.zip › Figure 6 Source Data 3/Figure 6B ╬▓-Actin Raw Data.pdf]

**Figure 6B**     YAP

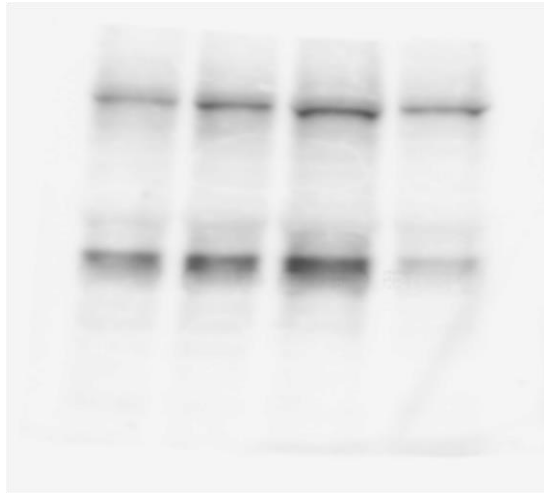

Supplement: Figure 6—source data 3. [file elife-95267-fig6-data3.zip › Figure 6 Source Data 3/Figure 6B YAP Raw Data.pdf]

**Figure6B    TAZ**

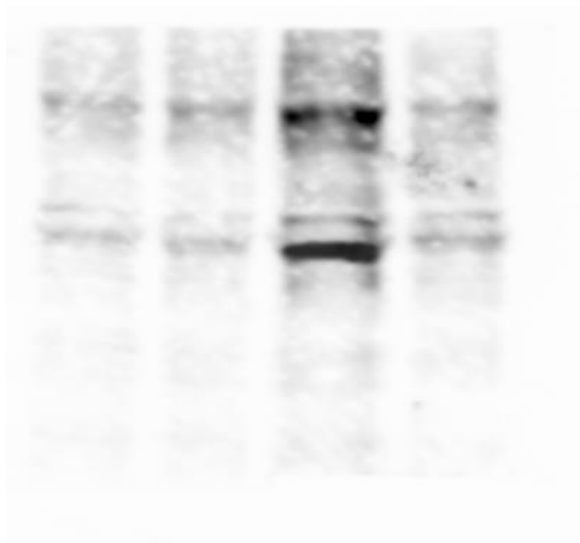

Supplement: Figure 6—source data 3. [file elife-95267-fig6-data3.zip › Figure 6 Source Data 3/Figure 6B TAZ Raw Data.pdf]
